# Dr. Edward Robinson Squibb

**Published:** 1900-12

**Authors:** 


Dr. Edward Robinson Squibb, of Brooklyn, died at his home October 29, 1900, of cardiac disease, aged 81 years. Dr. Squibb was early in his medical career in the naval service, finally attaining the rank of surgeon. He resigned early in the 60's, and established himself as a manufacturing chemist in Brooklyn. He did much to improve this important branch of chemical science, and the products of his laboratory were everywhere noted for purity and reliability.

Some years ago Dr. Squibb retired from active participation in the business, and it has since been conducted by his sons, Dr. Edward H. Squibb and Mr. C. H. Squibb.

